# Correction: Wu et al. 3D Thermal Network Supported by CF Felt for Improving the Thermal Performance of CF/C/Epoxy Composites. *Polymers* 2021, *13*, 980

**DOI:** 10.3390/polym14214628

**Published:** 2022-10-31

**Authors:** Xinfeng Wu, Yuan Gao, Tao Jiang, Lingyu Zheng, Ying Wang, Bo Tang, Kai Sun, Yuantao Zhao, Wenge Li, Ke Yang, Jinhong Yu

**Affiliations:** 1 College of Ocean Science and Engineering and Merchant Marine College, Shanghai Maritime University, Shanghai 201306, China; 2 School of Materials Science and Engineering, Central South University, Changsha 410083, China; 3 Key Laboratory of Marine Materials and Related Technologies, Zhejiang Key Laboratory of Marine Materials and Protective Technologies, Ningbo Institute of Materials Technology & Engineering, Chinese Academy of Sciences, Ningbo 315201, China

## Error in Table

In the original article [1], there was a mistake in Table 1. The volume fraction of the CF/C was wrongly displayed in the original table. The corrected Table 1 appears below. The authors apologize for any inconvenience caused and state that the scientific conclusions are unaffected. The original article has been updated.

## Figures and Tables

**Table 1:** The loading of the CF/C in the various CF composites.

Samples	CF/C [vol%]	CF/C [wt%]
CF/C/EP-1	11.3	17.0
CF/C/EP-1.5	15.8	23.3
CF/C/EP-2	22.8	32.2
CF/C/EP-2.5	25.3	35.2
